# Focal p53 protein expression and lymphovascular invasion in primary prostate tumors predict metastatic progression

**DOI:** 10.1038/s41598-022-08826-5

**Published:** 2022-03-30

**Authors:** William Gesztes, Cara Schafer, Denise Young, Jesse Fox, Jiji Jiang, Yongmei Chen, Huai-Ching Kuo, Kuwong B. Mwamukonda, Albert Dobi, Allen P. Burke, Judd W. Moul, David G. McLeod, Inger L. Rosner, Gyorgy Petrovics, Shyh-Han Tan, Jennifer Cullen, Shiv Srivastava, Isabell A. Sesterhenn

**Affiliations:** 1 Center for Prostate Disease Research, Murtha Cancer Center Research Program, Department of Surgery, Uniformed Services University of the Health Sciences, Bethesda, MD 20817 USA; 2 Henry M. Jackson Foundation for the Advancement of Military Medicine, Inc., Bethesda, MD 20817 USA; 3 Joint Pathology Center, Silver Spring, MD 20910 USA; 4 Urology Service, Walter Reed National Military Medical Center, Bethesda, MD 20852 USA; 5 Present Address: George Washington University Hospital, Washington, DC 20037 USA; 6 Present Address: Personal Genome Diagnostics, Baltimore, MD 21224 USA; 7 Present Address: Eli Lilly and Company, Indianapolis, IN 46285 USA; 8 Present Address: Infectious Disease Clinical Research Program, Bethesda, MD 20817 USA; 9 Present Address: Fort Sam Houston, San Antonio, TX 78234 USA; 10 Present Address: University of Maryland School of Medicine, Baltimore, MD 21201 USA; 11 Present Address: Duke University School of Medicine, Durham, NC 27710 USA; 12 Present Address: Department of Urology, Inova Fairfax Hospital, Fairfax, VA 22031 USA; 13 Present Address: Case Comprehensive Cancer Center, Case Western Reserve University, Cleveland, OH 44106 USA; 14 Present Address: Department of Biochemistry and Molecular and Cell Biology, Georgetown University School of Medicine, Washington, DC 20057 USA; 15 Division of Genitourinary Pathology, Joint Pathology Center, 606 Stephen Sitter A venue, Silver Spring, MD 20910 USA

**Keywords:** Cancer, Urological cancer, Prostate cancer

## Abstract

*TP53* is one of the most frequently altered genes in prostate cancer. The precise assessment of its focal alterations in primary tumors by immunohistochemistry (IHC) has significantly enhanced its prognosis. p53 protein expression and lymphovascular invasion (LVI) were evaluated for predicting metastatic progression by IHC staining of representative whole-mounted prostate sections from a cohort of 189 radical prostatectomy patients with up to 20 years of clinical follow-up. Kaplan–Meier survival curves were used to examine time to distant metastasis (DM) as a function of p53 expression and LVI status. *TP53* targeted sequencing was performed in ten tumors with the highest expression of p53 staining. Nearly half (49.8%) of prostate tumors examined showed focal p53 expression while 26.6% showed evidence of LVI. p53(+) tumors had higher pathologic T stage, Grade Group, Nuclear Grade, and more frequent LVI. p53 expression of > 5% and LVI, individually and jointly, are associated with poorer DM-free survival. *TP53* mutations were detected in seven of ten tumors sequenced. Four tumors with the highest p53 expression harbored likely pathogenic or pathogenic mutations. High levels of p53 expression suggest the likelihood of pathogenic *TP53* alterations and, together with LVI status, could enhance early prognostication of prostate cancer progression.

## Introduction

Prostate cancer (PCa) is the most common cancer and the second leading cause of cancer death among American men^1^. Although the presence of distant metastases at the time of diagnosis is rare, the likelihood of disease progression creates a need for predictive and prognostic biomarkers. Several potential molecular markers have been evaluated in radical prostatectomy (RP) specimens by immuno-histochemistry (IHC)^2^, and in biopsy tissues by multiplex immunofluorescence^3^, but none have become widely used in clinical practice. Recent genomic analyses support the association of *TP53* mutations with the initiation and progression of diverse neoplasms^4,5^. In both localized and advanced prostate cancers, *TP53* is one of the most frequently altered genes^6,7^. Approximately 6–7% of primary tumors carry *TP53* missense, frameshift, or truncation mutations, and at least 1% have homozygous deletions^7,8^. Genomic analysis of non-indolent localized PCa revealed *TP53* to be one of six genes with > 2% somatic single nucleotide variants (SNVs)^9^. The higher frequency of *TP53* lesions in localized cancers suggests that they arise relatively early in disease progression. In advanced PCa, the rate of *TP53* mutations becomes significantly enriched, approaching 40% SNVs, and 10% homozygous deletions or genomic rearrangements^10–12^.

*TP53* mutations that increase the stability and half-life of mutant proteins in cancer cells and enhance protein detection by IHC^4,13,14^ characterize a subgroup of biologically aggressive prostate cancers with high risk of progression after prostatectomy. Multiple studies have reported a correlation between IHC detection of p53 and PCa progression^14–20^. DNA sequencing of p53 positive (p53(+)) prostate tumors from 16 patients by Griewe et al., found a 69% correlation between p53 expression and *TP53* mutation^21^. Schlomm et al., reported a low frequency of p53(+) tumors (2.5% or 62/2514) by IHC in a tissue microarray from RP specimens, of which 47% (29/62) were found to harbor mutations associated with more aggressive disease^18^. In another screen of two overlapping RP patient cohorts with primary prostate tumors, Guedes et al. reported a high positive predictive value (84%) of p53 nuclear staining for underlying *TP53* missense mutation^4^. Importantly, in a single-patient longitudinal study, p53(+) metastatic lesions that developed years post-surgery could be traced to a low-grade p53(+) tumor focus in the primary tumor^22^. These findings emphasize the biological impact of focal *TP53* alterations in the clonal progression of PCa and support p53 IHC detection in primary PCa as a surrogate indicator of *TP53* missense mutations.

In PCa, lymphovascular invasion (LVI) has been shown to be associated with aggressive disease and poor prognosis, as defined by reduced biochemical recurrence (BCR) progression-free survival^23–25^, increased risk of PCa-specific mortality^26,27^ and other pathologic features of aggressive disease^28,29^. LVI has been evaluated with either *TP53* mutation or p53 expression in association with gastric^30^, colorectal^31^, bladder^32^ and breast cancer^33^ prognosis, but not PCa. This study examines the role of p53 protein expression and LVI in predicting distant metastasis (DM) in a RP cohort with long-term follow-up. We further explored the combined effect of p53 expression and LVI status on DM-free survival. To determine if tumors with higher p53 expression also harbor *TP53* mutations, targeted *TP53* sequencing was performed on a subset of prostate tumors with the highest percent of p53 expression.

## Methods

### Study design, population, and clinical assessment

Prostate specimens and clinical-pathologic data were collected from patients undergoing treatment at the Walter Reed National Military Medical Center (WRNMMC) from 1993 to 2013 who provided written informed consent for the use of all data and biospecimens obtained. Patients who had biopsy positive, organ-confined PCa and underwent RP as primary treatment (≤ 6 months post-diagnosis) were included, and those who underwent neo-adjuvant hormonal therapy were excluded. Archived, whole-mounted RP specimens from 50 patients who developed DM at least one year following diagnosis and from 139 patients without evidence of BCR or DM after at least 10 years follow-up, were analyzed. The presence of distant metastases was ascertained by the review of each patient’s complete radiographic scan history that included bone scan, computed tomography (CT), positron emission tomography (PET), as well as pelvic and bone magnetic resonance imaging (MRI). Subjects who reached the end of the study period without evidence of PCa metastases, had their last known follow-up, or died without evidence of metastasis before the end of the study period (December 31, 2013), were defined as non-metastatic. This work was approved by the Institutional Review Boards of WRNMMC, the Uniformed Services University of the Health Sciences (USU), and the Joint Pathology Center (JPC) (Protocol number DBS.2020.110).

### Immunohistochemistry and pathologic assessment

Preparation and histologic evaluation of whole-mounted RP specimens were performed as previously described^34,35^. Adjacent, four-micron sections from a representative tissue block containing the index tumor were stained with hematoxylin and eosin (H&E), anti-p53 mouse monoclonal antibody (DO-7, Biocare Medical, Pacheco, CA), and anti-podoplanin antibody (D2-40, Biocare Medical) to identify p53 and lymphatic vessels, respectively. Slides were reviewed using the 2014 International Society of Urological Pathology (ISUP) guidelines^36^ by a single genitourinary pathologist (I.A.S.), who was blinded to clinical outcomes. The p53 status in index tumors was scored as positive based on the detection of nuclear p53 staining, percent area stained, and staining intensity. Cells were recorded as p53 positive or p53(+), when brown chromogen (3,3’-Diaminobenzidine (DAB)) used to stain the DO-7 antibody was detected in the nuclei of any tumor cells, and as negative or p53(−) in the absence of any nuclear staining. Occasional tumor cells with exclusive cytoplasmic staining of any intensity were considered “negative”. Percentage of p53(+) staining was estimated as the area of p53(+) tumor cells with nuclear staining divided by total index tumor area^16^, which was categorized as 0%, 1–5%, and > 5% p53(+) expression. p53 staining intensity was also quantified as 1 + (light), 2 + (medium), and 3 + (maximum) intensity^37^. An independent pathologist review of p53 staining was performed by A.P.B. Findings were presented as percentage of p53 expression. LVI status was recorded as positive or LVI(+), when tumor cells were present within spaces lined by lymphovascular endothelium with characteristic podoplanin staining, and as negative or LVI(−) in the absence of any staining.

### Statistical analysis

Overall and p53-stratified (0%, 1–5%, > 5%) distributions for patient demographics, as well as clinical and pathologic features were compared using Student’s T-test for continuous variables and Chi-square and ANOVA tests for categorical variables. Fisher’s exact test was used when > 20% of expected cell counts had less than five observations. Unadjusted Kaplan–Meier (KM) estimation curves were used to examine time to DM as a function of p53 status. Log-rank test and its associated p-value are reported for KM models. Associations of p53 and LVI with DM-free survival were first evaluated independently and then jointly. Multivariable Cox Proportional Hazards analysis was used to model DM-free survival, controlling for demographic and pathologic factors. The assumption of proportional hazards was tested and confirmed for all KM and Cox models. All statistical tests were 2-sided (summary *α-*error = 0.05), and the decision rule was based on p < 0.05. All statistical analyses were performed using SAS version 9.4.

### TP53 mutation analysis

Index tumors were scraped from two adjacent whole-mounted FFPE sections derived from ten cases that were selected for targeted sequencing. Library preparations and sequencing reactions were conducted at GENEWIZ, Inc. (South Plainfield, NJ). Gene-specific primers targeting the *TP53* CDS were multiplexed into three pools. A sequencing library was prepared using the NEBNext Ultra DNA Library Preparation Kit (New England Biolabs, Ipswich, MA), validated using an Agilent TapeStation (Agilent Technologies, Palo Alto, CA), and quantified by Qubit (Invitrogen, Thermo Scientific, Waltham, MA) and real-time PCR (Applied Biosystems, Carlsbad, CA). Multiplexed DNA libraries were loaded on an Illumina MiSeq instrument (Illumina, San Diego, CA) for 2 × 150 bp paired-end sequencing. Image analysis and base calling were performed using MiSeq Control. Raw reads were aligned to the GRCh37 human reference genome using Burrows-Wheeler Aligner-mem. Samtools fixmate was used to correct any flaws in read-pairing introduced during alignment, and duplicate reads were marked using Picard. Alignments were subjected to base quality score recalibration, according to GATK best practices. Variants were identified using GATK Haplotype Caller and FreeBayes and annotated using the Ensembl Variant Effect Predictor toolset that included Sanger Catalogue of Somatic Mutations In Cancer (COSMIC) and ClinVar annotations from March 2021.

### Ethics approval and consent to participate

These prostate specimens and clinical-pathologic data in this study were collected from patients undergoing treatment at the WRNNMMC who provided written informed consent for their use. This work was approved by the Institutional Review Boards of the WRNMMC, USU, and JPC (Protocol number DBS.2020.110) and performed in accordance with the Declaration of Helsinki.

### Disclaimers

The contents of this publication are the sole responsibility of the author(s) and do not necessarily reflect the views, opinions or policies of the Henry M. Jackson Foundation for the Advancement of Military Medicine, Inc., the Uniformed Services University of the Health Sciences (USUHS), the Department of Defense (DoD) or the Departments of the Army, Navy, or Air Force, or any other agency of the US Government. The mention of trade names, specific commercial products, scientific instrumentation, or organizations is considered an integral part of the scientific endeavor and does not constitute endorsement or implied endorsement on the part of the author, DoD, or the US Government.

## Results

### Association of p53 expression and LVI with pathologic features

**Figure 1:**
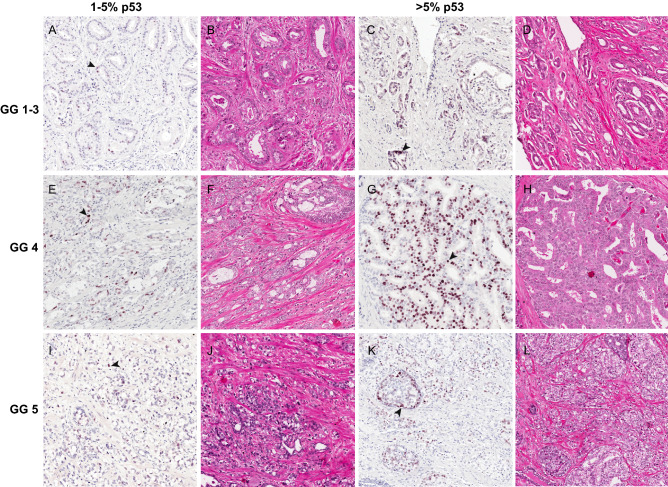
Representative p53 expression in index tumors clustered by tumor grade. p53 staining, detected at variable degrees of intensity between 1 and 5% (**A**, **E** and **F**) and > 5% (**C**, **G**, and **K**) and their corresponding sections stained with (**H**&**E**) are shown for each of the GG clusters: GG 1–3 (**A** to **D**), GG 4 (**E** to **H**), and GG 5 (**I** to **L**). Images were captured at 10X magnification. Arrowhead indicates individual cells stained by the p53 antibody.

**Figure 2:**
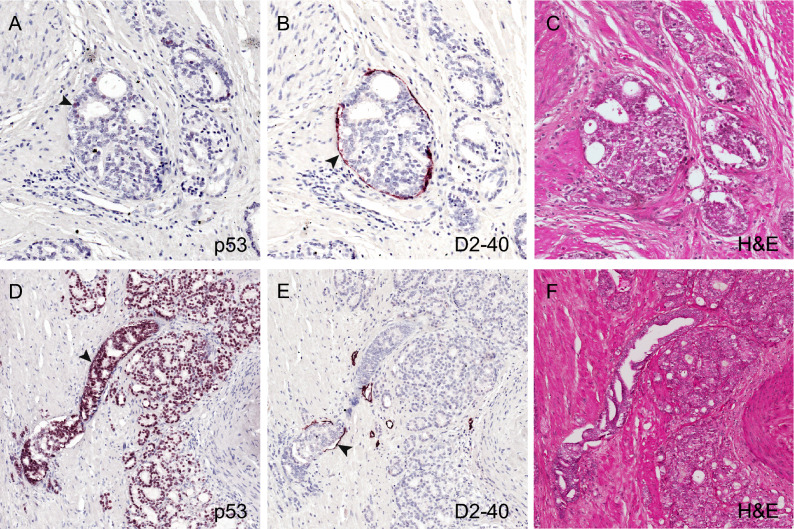
Representative p53 and lymphovascular staining in index tumors. p53 staining was detected at variable degrees of intensity between 1 and 5% in single cells (**A**) and in clusters of cells with greater than 5% (**D**) in relation to the area of the index tumor. The infiltration of tumor cells into lymphovascular spaces was confirmed by D2-40 IHC staining (**B**,**E**). The corresponding (**H**&**E**) images are shown in panel (**C**) and (**F**). Images were captured at 10X magnification. Arrowhead indicates individual cells stained by the p53 and D2-40 monoclonal antibodies.

Pathologic features of tumors in adjacent whole-mounted prostate sections that were immuno-stained with p53, podoplanin and H&E were evaluated together for p53 expression and LVI status. Representative cases with 1–5% and > 5% p53 staining for each of the three Grade Group (GG) clusters, GG1-3, GG4 and GG5, are shown in Fig. 1. The presence of both lymphovascular invasion and p53 staining in index tumors are represented by cases in Fig. 2. Cells with p53(+) nuclear staining appeared as isolated cells (Figs. 1A and 2A), in clusters (Fig. 1C,G,K, and Fig. 2D), or both (Fig. 1E,I). Focal p53(+) staining was present in 94 (49.8%) and absent in 95 (50.2%) men. Among patients with p53(+) nuclear staining, 40% exhibited 1–5% expression, most (80%) of which had about 1% p53 expression (Table 1). Among the 75 subjects with 1–5% p53 expression, we noted that 60 cases (80%) had 1% expression, four cases had 2% expression, three cases had 3% expression, and eight cases had 5% expression. The median of percentage p53 expression among the 19 subjects with > 5% p53 expression is 25%. Generally, most subjects at or below the threshold of 5% p53 expression showed either light (1 +) (40% or 30 cases) or medium (2 +) (55% or 41 cases) staining intensity, while only 5% (four cases) showed maximum (3 +) staining intensity. In contrast, most subjects with > 5% p53 expression showed 3 + intensity (58% or 11 cases), while the remaining cases showed either 2 + (32% or six cases) or 1 + (10% or 2 cases) staining intensity. Higher levels of p53(+) nuclear staining were associated with aggressive tumor pathologic features, as indicated by pathologic T stage (pT), GG, Nuclear Grade, and LVI.

**Table 1:** Patient demographic and clinico-pathologic features distributed across categories of percent p53 expression (N = 189).

Variable	All	Percent p53 expression			P-value
		0%	1–5%	> 5%	
**N (%)**	189	95 (50.3)	75 (39.7)	19 (10.1)	
**Age at radical prostatectomy (year)**					
Mean (SD)	60.4 (7.3)	60.5 (7.7)	59.5 (7.3)	62.7 (5.3)	0.2771
**PSA at diagnosis (ng/mL)**					
Median (range)	5.8 (0.4–94.2)	5.4 (0.4–88.7)	6.1 (0.7–94.2)	7.4 (1.1–38.9)	0.4219
**Follow up years**					
Median (range)	13.0 (1.6–21.0)	12.7 (2.0–20.3)	13.6 (2.5–20.6)	11.4(1.6–21.1)	0.0547
**Race**^**†**^					
Caucasian American	130 (69.9)	59 (63.4)	57 (77.0)	14 (73.7)	
African American	56 (30.1)	34 (36.6)	17 (23.0)	5 (26.3)	0.1526
**Pathological T stage**					
pT2	116 (61.4)	68 (71.6)	43 (57.3)	5 (26.3)	
pT3–4	73 (38.6)	27 (28.4)	32 (42.7)	14 (73.7)	**0.0007**
**Grade group (GG)**					
GG 1–3	85 (45.0)	52 (54.7)	30 (40.0)	3 (15.8)	
GG 4	52 (27.5)	19 (20.0)	28 (37.3)	5 (26.3)	
GG 5	52 (27.5)	24 (25.3)	17 (22.7)	11 (57.9)	**0.0013**
**Surgical margin**					
Negative	130 (69.2)	71 (74.7)	50 (67.6)	9 (47.4)	
Positive	58 (30.8)	24 (25.3)	24 (32.4)	10 (52.6)	0.0578
**Nuclear grade**^**§**^					
I	30 (16.0)	19 (21.2)	9 (12.0)	2 (11.1)	
II	138 (73.8)	69 (73.4)	59 (78.7)	10 (55.6)	
III	19 (10.2)	6 (6.4)	7 (9.3)	6 (33.3)	**0.0208**
**Lymphovascular invasion**^||^					
No (–)	138 (73.4)	82 (86.3)	48 (64.0)	8 (44.4)	
Yes (+)	50 (26.6)	13 (13.7)	27 (36.0)	10 (55.6)	** < 0.0001**
**Distant metastasis (DM)**					
No (–)	139 (73.5)	76(80.0)	57(76.0)	6 (31.6)	
Yes (+)	50 (26.5)	19 (20.0)	18 (24.0)	13 (68.4)	** < 0.0001**

Significant values (P-value < 0.05) are in bold.

^**†**^Three subjects who were neither Caucasian nor African American race were excluded.

^**§**^Two missing subject data due to treatment effect.

^||^One missing subject data due to capsular incision on whole-mount specimen; appropriate staging not possible.

Lymphovascular invasion was characterized by the infiltration of tumor cells within spaces lined by D2-40 stained lymphovascular endothelium (Fig. 2B,E), which confirmed features observed in the corresponding hematoxylin and eosin images (Fig. 2C,F, respectively). Evaluation of whole-mounted sections immuno-stained with D2-40 showed that 50 of 188 specimens (26.6%) were LVI(+) while 138 (73.4%) had no evidence of LVI. We observed that lymphovascular invasion was positively associated with p53 expression. Among p53(+) cases, 39.8% (37/93) were LVI(+) compared to p53(−) cases, where only 13.7% were LVI(+). Furthermore, more than half or 55.6% of patients with > 5% p53(+) expression were LVI(+) compared to 36% of patients with 1–5% p53(+) expression and 13.7% of p53(−) patients (Table 1; p < 0.0001). LVI(+) status was also associated with higher diagnostic PSA and pathologic features of more aggressive disease, including higher pT, GG, and Nuclear Grade, and positive surgical margin (Table 2). No association between p53 expression or LVI status with patient self-reported race was observed (Tables 1 and 2).

**Table 2:** Associations of lymphovascular invasion and p53 expression status with demographic and clinicopathological variables.

Variable	Lymphovascular invasion status		P value			
	LVI (–)	LVI (+)				
**N (%)**	138 (73.4)	50 (26.6)				
**Age at RP (year)**						
Mean (SD)	59.9 (7.7)	61.6 (6.3)	0.1400			
**PSA at diagnosis (ng/mL)**						
Median (range)	5.1 (0.4–94.2)	7.4 (1.1–38.9)	**0.0027**			
**Follow up years**						
Median (range)	13.0 (1.6–20.1)	12.7 (2.5–21.1)	0.6575			
**Race**						
Caucasian American	99 (73.3)	30 (60.0)				
African American	36 (26.7)	20 (40.0)	0.0796			
**Pathologic T stage**^**†**^						
pT2	107 (77.5)	8 (16.0)				
pT3–4	31 (22.5)	42 (84.0)	** < 0.0001**			
**Grade Group (GG)**^**†**^						
GG1–3	79 (57.2)	5 (10.0)				
GG4	33 (23.9)	19 (38.0)				
GG5	26 (18.8)	26 (52.0)	** < 0.0001**			
**Nuclear grade**						
I	27 (19.6)	2 (4.2)				
II	99 (71.7)	39 (81.3)				
III	12 (8.7)	7 (14.6)	**0.0187**			
**Surgical margin**						
Negative	107 (77.5)	23 (46.9)				
Positive	31 (22.5)	26 (53.1)	** < 0.0001**			
**Distant metastasis**						
No	118 (85.5)	20 (40.0)				
Yes	20 (14.5)	30 (60.0)	** < 0.0001**			

	p53 expression and lymphovascular invasion status					
	p53 0–5% & LVI(–)	p53 > 5% & LVI(–)	p53 0–5% & LVI(+)	p53 > 5% & LVI(+)	Total	p-value
**Metastasis**						
No	114 (87.7)	4 (50.0)	19 (47.5)	2 (18.2)	139 (73.5)	
Yes	16 (12.3)	4 (50.0)	21 (52.5)	9 (81.8)	50 (26.5)	** < 0.0001**

Significant values (P-value < 0.05) are in bold.

^**†**^Appropriate staging was not possible in one patient due to unavailability of capsular incision data.

### Focal p53 expression and LVI in RP specimens predict metastatic progression

**Figure 3:**
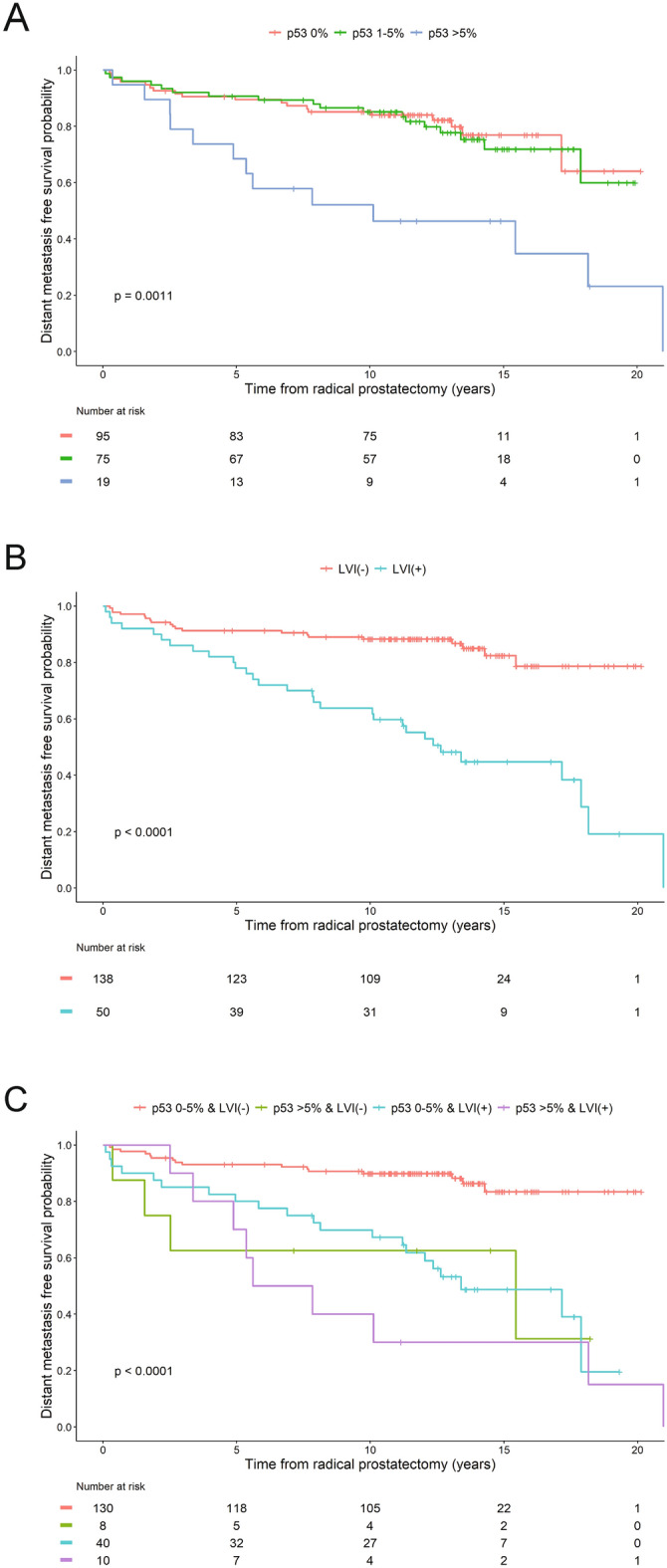
Product limit estimates for p53 and LVI with distant metastasis-free survival as endpoint events. Unadjusted Kaplan–Meier estimation curves showing models of time from surgery to DM-free survival as a function of p53 expression (**A**), LVI status (**B**), and combined p53 expression and LVI status (**C**), in index tumors as key independent study predictors for all patients (N = 189).

Distant metastasis developed in 68.4% of patients with > 5% p53(+) expression but only in 20% and in 24% of those without p53 or with 1–5% p53(+) expression, respectively (Table 1). Unadjusted univariable KM analysis showed that > 5% p53 expression was associated with significantly poorer DM-free survival (Fig. 3A). Separation in KM curves across levels of p53(+) expression occurred by three years post-RP. Notably, patients without detectable p53 expression and those with 1–5% p53(+) expression, had comparable DM-free survival outcomes.

The association between LVI status and DM revealed that 60% (30/50) of LVI(+) patients developed DM compared to 14.5% (20/138) of those without LVI (Table 2). In univariable KM analysis, LVI(+) status predicted significantly poorer DM-free survival (Fig. 3B) with separation in KM curves observed early in subject follow-up. Further evaluation of the association of p53 protein expression together with LVI was performed by merging the 0% and 1–5% p53 expression groups into one 0–5% p53(+) category. KM analysis of the joint roles of p53 and LVI status showed the poorest DM-free survival in patients with > 5% p53(+) and LVI(+) status, among which 81.8% developed DM (Fig. 3C; Table 2).

### Multivariable Cox proportional hazard models predict distant metastasis-free survival

Multivariable Cox Proportional hazards analysis was used to examine independent and joint roles of p53 expression and LVI status, together with patient’s age at RP and race, on DM-free survival (Table 3). Three approaches were used: (1) Model One shows a strong correlation between p53 expression of > 5% and increased risk of DM, which increases the hazard for this event by three-fold (hazard ratio (HR) = 3.173; p = 0.0006). (2) Model Two shows that both > 5% p53 expression and LVI(+) are independent predictors of shorter time to DM. When other covariates were held constant, the hazard for DM increased by two-fold (HR = 2.224; p = 0.0225) for > 5% p53 expression, and by four-fold (HR = 4.053; p < 0.0001) for LVI(+) status (3) In Model Three, the joint analysis of p53 expression and LVI status showed that p53 expression of > 5% and LVI(+) status confer incremental risk for DM: the hazard for this event increased by 4.4-fold-fold (HR = 4.428; p = 0.0091) based on > 5% p53 expression alone, and by almost five-fold (HR = 4.839; p < 0.0001) based on LVI(+) status alone, but together they increased the risk by almost eight-fold (HR = 7.976; p < 0.0001). This suggests a strong mutual and additive impact of higher p53 expression and LVI(+) status on DM-free survival. By contrast, in all three models, after adjusting for the patient’s race and p53 expression levels or LVI(+) status, an additional year of age at RP was shown to induce hazards of DM only by a factor of 1.03 to 1.04 (or 3–4%). Thus, increasing age at RP contributes little to the difference in the risk of DM. Likewise, after adjusting for the patient’s age and p53 expression levels, the patient’s race had no significant effect on the hazard of DM. The poorest DM-free survival outcomes were observed among patients who exhibited both > 5% p53(+) and LVI(+) status. In all models, significant correlations between GG and pT with p53 and LVI status prevented their inclusion in multivariate models. Moreover, too few patients were observed in lower GG (1–2) and stage categories.

**Table 3:** Multivariable cox proportional hazards analysis predicting distant metastasis-free survival^1^.

Variable	Model one^2^			Model two^3^			Model three^4^		P value
	HR	95% CI of HR	P value	HR	95% CI of HR	P value	HR	95% CI of HR	
**Age at RP** (years)	1.042	0.999–1.088	0.0577	1.03	0.986–1.077	0.1788	1.035	0.989–1.082	0.1356
**Race**									
Caucasian American	1			1			1		
African American	0.795	0.420–1.507	0.4821	0.561	0.289–1.092	0.0888	0.603	0.308–1.181	0.1404
**Percent p53 expression**^**5**^									
0–5%	1			1			NA		
> 5%	3.173	1.638–6.146	0.0006	2.224	1.119–4.420	0.0225	NA	NA	NA
**LVI status**									
LVI(–)	NA			1			NA		
LVI(+)	NA	NA	NA	4.053	2.217–7.409	< .0001	NA	NA	NA
**p53 expression and LVI status**									
0–5% p53 & LVI(–)	NA			NA			1		
> 5% p53 & LVI(–)	NA	NA	NA	NA	NA	NA	4.428	1.448–13.537	0.0091
0–5% p53 & LVI(+)	NA	NA	NA	NA	NA	NA	4.839	2.511–9.326	< 0.0001
> 5% p53 & LVI(+)	NA	NA	NA	NA	NA	NA	7.976	3.304–19.252	< 0.0001

LVI = lymphovascular invasion; RP = Radical Prostatectomy; HR = Hazards Ratio; CI = Confidence Interval.

^1^Due to oversampling for advanced pathologic stage and grade, there was little to no heterogeneity with respect to these subject features across outcome status, preventing their inclusion in the multivariable model.

^2^Model One: main effect of p53 is entered as key independent predictor of distant metastasis-free survival.

^3^Model two: both main effect of p53 and LVI are entered as key independent predictors of distant metastasis-free survival.

^4^Model three: a cross-tabulation of p53 and LVI is entered as key independent predictor of distant metastasis-free survival.

^5^Percent p53 expression was dichotomized, based on results of KM analysis showing equivalent distant metastasis-free survival probabilities for groups with 0 and 1–5% p53 expression.

### Association between *TP53* mutations and p53 expression or LVI status

Tumor specimens of ten patients with the highest p53 staining (20% to 90%) were selected for targeted TP53 sequencing. The high-depth coverage achieved by *TP53* targeted sequencing allowed SNVs to be detected at relatively higher alternate allele frequencies of 0.11 to 0.51. Almost all patients sequenced for *TP53* developed aggressive disease, represented by GG 4 or 5, pT3, or DM (Table 4). At least one missense or nonsense *TP53* mutation was detected in seven patients, and two mutations were detected in one patient. *TP53* mutations were detected in all four patients who had both high (> 30%) expression of p53 and LVI(+) status. Interestingly, in all four patients who had both high expression of p53 and LVI(+) status, the *TP53* mutations detected were either likely pathogenic or pathogenic alterations, which were also among the most recurrent *TP53* mutations in the COSMIC database. In agreement with results showing an association of LVI(+) status with poorer DM-free survival (Table 2 and Fig. 3B), all five subjects sequenced who were LVI(+) further developed DM. The number of cases sequenced, however, were too small to indicate any association of LVI positivity with specific mutational status.

**Table 4:** Clinico-pathologic features and *TP53* mutations for a subset of ten patients with p53(+) ≥ 20%.

p53 expression status and clinico-pathologic features									Single nucleotide variant (SNV)								
Patient	%p53	p53 intensity	LVI	pT	GG	NG	SM	DM	Chr,Site,Ref,Alt	NT	AA	DP	ALT	ClinVar Interpretation	COSMIC AnNotation	COSMIC count	Refs
1^§^	90	3	Yes	T3a	5	III	Pos	Yes	17,7578235,T,C; 17,7577094,G,C	c.614A > G; c.844C > G	p.Y205C; p.R282G	4099; 2651	0.21; 0.11	Likely pathogenic; Pathogenic	COSM43947; COSM10992	137;55	^9,47^
2	70	3	Yes	T3b	5	II	Pos	Yes	17,7574018,G,A	c.1009C > T	p.R337C	4837	0.31	Pathogenic/ Likely pathogenic	COSM11071	138	^12,48–50^
3	70	3	No	T3a	4	III	Neg	Yes	17,7577090,C,G	c.848G > C	p.R283P	2011	0.22	Uncertain significance	COSM10743	44	
4	70	2	Yes	T3a	5	II	Neg	Yes	17,7576855,G,A	c.991C > T	p.Q331*	1952	0.27	Pathogenic	COSM11354	98	^12,51,52^
5	30	3	Yes	T3b	5	III	Pos	Yes	17,7577120,C,A	c.818G > T	p.R273L	4919	0.28	Pathogenic	COSM10779	235	^39,52,53^
6	25	3	No	T3b	5	II	Pos	No	17,7578262,C,A	c.587G > T	p.R196L	3460	0.22	Uncertain significance	COSM45444	8	
7	25	2	No	T2b	1	II	Neg	No									
8	20	3	Yes	T3b	5	II	Pos	Yes									
9	20	3	No	T3a	5	III	Neg	Yes	17,7577551,C,A	c.730G > T	p.G244C	1466	0.51	Uncertain significance	COSM11524	82	
10	20	2	Yes	T3b	4	II	Pos	No									

LVI = Lymphovascular Invasion; pT = Pathologic Stage; GG = Grade Group; NG = Nuclear Grade; SM = Surgical Margin; DM = Distant Metastasis; NT = Nucleotide change; AA = Amino Acid change; DP = Depth of Coverage; ALT = Frequency of alternate allele; Pos = positive; Neg = Negative.

^§^Two distinct *TP53* mutations were detected in patient 1 who exhibited 90% p53 protein expression.

## Discussion

In this study, p53 expression and LVI status were examined as key independent predictors of DM. High p53 expression was significantly associated with DM, the frequency of which was three-fold higher in patients with > 5% p53(+) compared to patients with 0–5% p53(+). By stratifying the data at > 5% cut-off, we were able to distinguish between two clinically relevant p53(+) populations: patients with > 5% p53(+) have significantly shorter DM-free survival than those with 0–5% p53(+). Likewise, the presence of LVI is associated with higher frequency of DM. LVI(+) patients developed DM at a rate that was four-fold higher than those without LVI. Further analysis by unadjusted univariable KM further confirmed that LVI(+) status significantly predicts poorer DM-free survival. Subsequent combined examination of p53 expression and LVI by multivariable analyses showed that together, they exerted an additive increase in risk for DM.

Although multiple studies have shown an association between p53 expression and *TP53* mutation^4,18,21,22^, inconsistencies were noted by others. These discrepancies could be attributed to limitations of the IHC assay, including antibodies used for detection^38,39^, or to study cohort selection^40^. The focality of *TP53* alterations in primary PCa can lead to differences in IHC interpretations or DNA sequencing assays^4,16,21^. Since p53 IHC detection depends on the increased half-life of mutant proteins, proteins with destabilizing mutations may escape detection^41^. The lower frequency of *TP53* mutations in localized prostate cancers could reduce the likelihood for finding an association with increased p53 expression^18,40^. Since p53 nuclear accumulation is far more frequent in higher grade carcinomas, performing IHC on all primary prostate cancers at diagnosis is unlikely to establish the expected association^4^. By contrast, this study is designed to focus on defining the association of p53 expression and LVI status with DM. Hence, the proportion of subjects with advanced stage (pT3-4) and grade (GG 4 & 5) is greater in this cohort than subjects who undergo RP without neoadjuvant therapy in the general population. One advantage of this study is the availability of primary PCa specimens with associated long-term follow-up (median = 13 years) data obtained from an equal-access military treatment facility. The greater proportion of patients with aggressive disease, who undergo RP without neo-adjuvant therapy in this cohort compared to patients in the general population allowed us to demonstrate the striking association between both focal p53(+) expression and LVI(+) status, and the development of DM. Furthermore, the use of whole-mounted prostate sections augmented the comprehensive evaluation of p53 expression and LVI in index tumors compared to using tissue microarrays^18,42^ or biopsy specimens^38,43^. Lastly, the concordant scores of percent p53 expression between two independent pathologists further validated that this approach was more reproducible than by staining intensity alone (92% vs. 72%, respectively).

Based on earlier reports that p53 positive tumors were likely due to mutations that increased the half-life of the p53 protein, we hypothesized that tumors with the highest percentage p53 expression would have a higher probability of harboring TP53 mutations. To test this notion, we selected ten cases with the highest percentage of p53 expression for targeted *TP53* sequencing. *TP53* mutations were detected in seven of ten cases analyzed. These mutations, which include the most recurrent hotspot at Arginine 273, were previously reported in advanced or metastatic PCa and annotated in COSMIC^44^ and ClinVar^45^ databases. Four patients harboring likely pathogenic or pathogenic *TP53* mutations had aggressive disease represented by GG 5, pT3 and LVI(+) tumors that progressed to DM. Consistent with earlier reports, concordance between p53 staining and the presence of pathogenic *TP53* mutations further supports the prognostic utility of IHC detection as a surrogate read-out for *TP53* mutations^4,21^.

Although LVI is known to be associated with aggressive disease and poor prognosis in PCa, no direct comparison to *TP53* mutations or its protein expression has been performed^23–26^. One study reported ERG(+) tumors had higher LVI and lower p53 expression in ERG(+) tumors, but no significant association was detected due to sample size (N = 51)^46^. The most striking finding of this study was that 81.8% of patients with both > 5% p53(+) expression and LVI(+) status developed DM after an extended follow-up period, while 87.7% patients with LVI(−) and < 5% p53(+) were DM-free. The joint interpretation of these two variables is underscored by three key findings: (1) p53(+) expression of > 5% strongly correlates with LVI(+) status, (2) multivariate analysis suggests p53 expression and LVI status to be additive, and (3) tumors with lower p53 expression of 1–5% may represent less aggressive disease as these patients have DM-free survival outcomes that are remarkably similar to patients without p53 staining.

## Conclusion

Our findings validated the association between pathogenic *TP53* mutations and higher p53 expression, which support the IHC staining of p53 as a substitute for detecting *TP53* mutations. Primary prostate tumors with combined focal p53(+) of > 5% and LVI(+) status are highly predictive of future DM and should be classified as highly aggressive tumors. This subset of patients may require a more rigorous treatment plan and follow-up protocol. Taken together, determination of p53 expression and LVI status in primary PCa has promising potential to improve prognostication and early prevention of metastatic progression.

## Data Availability

The datasets generated and analyzed in this study are not publicly available due to restrictions imposed by the current IRB protocol but can be made available from the corresponding author upon approval of a separate IRB protocol allowing for their subsequent use.
